# Real-world treatment-transition pathways and dosing patterns of insulin degludec/liraglutide in Chinese adults with type 2 diabetes

**DOI:** 10.1016/j.metop.2026.100497

**Published:** 2026-09-15

**Authors:** Jie Zhang, Weihao Wang, Fuli Man, Xiaoxia Wang, Qi Pan, Lixin Guo

**Affiliations:** Department of Endocrinology, Beijing Hospital, National Center for Gerontology, National Clinical Research Center for Gerontology, The Key Laboratory of Geriatrics of NHC, Institute of Geriatric Medicine, Chinese Academy of Medical Sciences, Beijing, PR China

**Keywords:** GLP-1RA, Type 2 diabetes, Insulin, Degludec, Liraglutide

## Abstract

**Background and aims:**

To describe real-world treatment-transition pathways, early IDegLira dosing patterns, treatment simplification, and observed short-term clinical outcomes among Chinese adults with type 2 diabetes (T2D).

**Methods:**

This multicenter, non-interventional, retrospective observational registry included 1499 adults with T2D who initiated IDegLira in routine practice across 13 provinces and regions in China from April 2022 to December 2023. The five mutually exclusive prior-treatment pathways were non-injectable therapy, basal insulin, multiple daily injections (MDI), insulin plus GLP-1RA, and GLP-1RA-based therapy. Treatment decisions were physician-directed. Eligible patients had documented continuous use of once-daily IDegLira (the fixed-ratio combination [FRC]) for at least 3 months. Early dosing was defined as the starting dose, dose titration, and dose documented at approximately 12 weeks. Exploratory linear models estimated crude and covariate-adjusted between-pathway differences in paired HbA1c and body-weight change.

**Results:**

Baseline HbA1c was available for 1374 patients (9.05 ± 1.78%) and week-12 HbA1c for 498 patients (7.44 ± 2.29%). Among 480 patients with paired baseline and week-12 HbA1c data (all enrolled in the final study period), mean HbA1c decreased from 9.11 ± 2.09% to 7.45 ± 2.28% (mean change −1.66 ± 2.23 percentage points; P < 0.001). Among the week-12 evaluable population, 21.08%, 47.39%, 81.73%, and 89.36% had HbA1c ≤ 6.5%, <7.0%, <7.5%, and <8.0%, respectively. Among 487 patients with paired baseline and week-12 body-weight data (all from the final study period), mean body weight decreased from 75.48 ± 11.12 to 72.72 ± 10.56 kg (mean change −2.76 ± 4.93 kg). In exploratory adjusted models, HbA1c reductions were smaller after basal insulin (mean difference +0.82 percentage points, 95% CI 0.37 to 1.27) and MDI (+1.25, 95% CI 0.53 to 1.98) than after non-injectable therapy, whereas no overall adjusted pathway association was observed for body-weight change (global P = 0.606). The mean starting dose was 14.46 ± 4.06 U and the mean week-12/current dose was 16.72 ± 4.55 U among available cases. Prandial insulin discontinuation was recorded in 269/301 MDI patients (89.37%); concurrent paired HbA1c and systematic post-initiation safety data were unavailable for this subgroup.

**Conclusions:**

This study describes real-world treatment-transition and dosing patterns for IDegLira across heterogeneous prior therapies. Glycemic findings are based on the week-12 evaluable population (498 of 1499 patients, all enrolled in the final study period); among 480 patients with paired baseline and week-12 HbA1c data, mean HbA1c decreased by 1.66 percentage points. The study had no comparator group and no basis for causal inference. The findings are descriptive and hypothesis-generating, and may inform clinical discussions and future comparative studies.

## Highlights

• Real-world IDegLira treatment-transition patterns were described in 1499 Chinese adults with type 2 diabetes.
• Week-12 HbA1c was 7.44% among 498 patients with available data.
• HbA1c decreased by 1.66 percentage points among 480 patients with paired measurements.
• Prandial insulin discontinuation was recorded in 269/301 MDI patients (89.37%).
• Findings were descriptive and hypothesis-generating; no causal inference was possible.

## 1 Introduction

China has one of the largest national populations of adults living with diabetes, and this absolute burden is projected to increase further as the population ages [[1], [2], [3]].

Population-level studies have reported differences in body composition, age at onset, and diabetes phenotypes between East Asian and European-ancestry populations, although substantial heterogeneity exists within each population [[4], [5], [6], [7], [8]]. These population-level differences provide context for interpreting routine-care prescribing in China but do not imply that treatment response to IDegLira is determined by ethnicity.

As T2D progresses, treatment may be intensified from non-injectable therapy to injectable regimens. IDegLira combines basal insulin degludec and liraglutide in a fixed-ratio, once-daily formulation; 1 U contains 1 U insulin degludec and 0.036 mg liraglutide [[9], [10], [11], [12], [13]]. The contribution of liraglutide within the fixed-ratio combination has been evaluated in a component study [14]. The number of injections required depends on the prior regimen and the concomitant treatment plan.

Randomized IDegLira trials included selected insulin-naive and insulin-experienced populations and used predefined titration algorithms and protocolized follow-up [[15], [16], [17], [18], [19], [20], [21], [22]]. Related trials of basal-insulin/GLP-1RA intensification provide additional context [23,24]. These trials do not fully describe heterogeneous prior-treatment pathways, physician-directed initiation, treatment simplification, or reimbursement-related practice patterns in routine Chinese care.

IDegLira has been approved in China and was included in the National Reimbursement Drug List during the study period. Real-world uptake may nevertheless be influenced by patient selection, prescribing habits, drug availability, healthcare setting, and reimbursement. Existing and recent real-world studies have reported overall outcomes, treatment persistence, switching from basal or basal-bolus insulin, and regimen simplification, whereas less is known about how these patterns vary across a broader set of prior-treatment pathways in Chinese practice [[25], [26], [27], [28], [29], [30]].

This study therefore aimed primarily to describe treatment-transition pathways and early IDegLira dosing patterns in routine Chinese care. Secondary objectives were to describe week-12 HbA1c status among evaluable patients, weight and glucose measures, treatment simplification, concomitant medication changes, and temporal differences across observation periods. Post hoc exploratory analyses estimated associations between prior-treatment pathway and paired HbA1c and body-weight changes. Early dosing was defined as the starting dose, titration during the first approximately 12 weeks, and the dose recorded at follow-up.

## 2 Subjects, materials and methods

### 2.1 Study design and population

This multicenter, non-interventional, retrospective observational registry analyzed records from 1499 adults with T2D who initiated IDegLira in routine clinical practice across 13 province-level regions in China (Beijing, Tianjin, Chongqing, Henan, Shandong, Jiangsu, Zhejiang, Fujian, Guangdong, Liaoning, Qinghai, Shaanxi, and the Xinjiang Uygur Autonomous Region) between April 2022 and December 2023. Research-site selection and training were conducted by Ipsos Healthcare. Across the three recruitment periods, site selection included 736 hospitals, comprising 176 secondary-level hospitals (23.9%) and 560 tertiary-level hospitals (76.1%); the sampling plan maintained an urban-to-county ratio of 2:1. Participating physicians were senior attending physicians or above, and no physician contributed more than three enrolled patients (Supplementary Table 2).

Eligible patients were diagnosed with T2D according to the WHO 1999 criteria as documented in routine medical records [31], were 20–80 years of age, had not previously received IDegLira, and had documented continuous use of once-daily IDegLira (the fixed-ratio combination [FRC]) for at least 3 months with routine-care information available at or after this minimum duration. The 3-month requirement represented documented treatment exposure and follow-up availability, not a protocol-mandated visit or common titration schedule. Patients were excluded if they were participating in or had completed another clinical trial within the previous three months, had a positive human chorionic gonadotropin test, or could not comply with protocol contraception requirements; these were study eligibility criteria related to reproductive safety.

Treating physicians identified potentially eligible patients from outpatient clinics and wards and completed a standardized questionnaire using medical-record information and routine follow-up documentation. The analytic cohort was constructed from available records meeting the eligibility criteria during the study periods. Site training and questionnaire-completion procedures were coordinated by Ipsos Healthcare. The questionnaire captured baseline regimen, IDegLira initiation, dose information, concomitant glucose-lowering treatment, and available follow-up measurements. No formal independent inter-rater validation or duplicate data entry was documented; the cleaned database underwent range and consistency checks. The requirement for written informed consent was waived by the Ethics Committee of Beijing Hospital for this non-interventional study using routine clinical data. The central ethics approval was obtained on May 3, 2023 (protocol 2023BJYYEC-087-01).

The index date was the date of IDegLira initiation. Baseline referred to the pre-initiation assessment documented in routine care; follow-up referred to the available routine-care record at or after the minimum 3-month continuous-use period (approximately 12 weeks), without a protocol-mandated 10- to 14-week window. IDegLira dose was recorded in units at initiation and available follow-up; initiation, titration, concomitant medication changes, and discontinuation were determined by treating physicians without a common investigator-assigned algorithm. Treatment simplification was operationalized as documented discontinuation of prandial insulin among patients transitioning from MDI during available follow-up; it was not a validated composite endpoint. Reasons for IDegLira discontinuation were not systematically captured, so discontinuation was not analyzed as a separate outcome. Standardized post-initiation hypoglycemia, adverse-event, and serious-adverse-event data were not systematically available across all periods and were not analyzed.

The main analysis described week-12 HbA1c level and target attainment among patients with an available week-12 HbA1c measurement. Baseline HbA1c was summarized separately. Baseline HbA1c for the final study period was recovered after an interim unmapped baseline field had limited earlier extracts; paired baseline–week-12 HbA1c values were available for 480 patients, and the within-patient change was summarized descriptively for this paired population. Paired HbA1c data were not available for the earlier study periods, whose questionnaire did not capture follow-up HbA1c. Secondary analyses included fasting and postprandial glucose, body weight, BMI, IDegLira dose, treatment simplification, concomitant medication changes, and temporal patterns. The HbA1c thresholds (≤6.5%, <7.0%, <7.5%, and <8.0%) were prespecified descriptive thresholds applied uniformly to the evaluable week-12 population and were not intended to replace individualized treatment targets. Baseline characteristics of patients with and without week-12 HbA1c data were compared using two-sided Welch's t-tests (or Mann–Whitney U tests for skewed distributions) and chi-square tests (Supplementary Table 6).

Additional analyses described real-world IDegLira use, including patient characteristics, dosing patterns, concomitant medication changes, insurance category, baseline treatment regimen, and differences across observation periods (2022, 2023H1, and 2023H2).

### 2.2 Prior-treatment pathway definitions

Prior-treatment pathways were defined from the single physician-reported anti-hyperglycemic regimen recorded immediately before IDegLira initiation: (1) non-injectable therapy: oral glucose-lowering drugs (OADs) only or no pharmacotherapy; (2) basal insulin: basal insulin with or without OADs; (3) multiple daily injections (MDI): premixed insulin, co-formulated dual insulin, prandial (bolus) insulin, or continuous subcutaneous insulin infusion (insulin pump), with or without OADs; (4) insulin plus GLP-1RA: any regimen containing both insulin and a GLP-1RA; and (5) GLP-1RA-based therapy: a GLP-1RA with or without OADs and without insulin. Patients whose recorded regimen met criteria for more than one category were assigned using a fixed priority order (insulin plus GLP-1RA, then GLP-1RA-based therapy, then MDI, then basal insulin, then non-injectable therapy), so that the five pathways were mutually exclusive and exhaustive. Every patient was assigned to exactly one pathway. Prior-treatment duration was not captured consistently and was not used for classification.

### 2.3 Statistical analyses

Data analysis was performed using SAS version 9.4 (SAS Institute, Cary, NC, USA); the post hoc pathway-outcome models requested during peer review were implemented in Python 3.9 using statsmodels 0.14.6. Available-case analyses were used separately for each endpoint; no imputation was performed. Continuous variables are presented as mean ± SD and, where useful, median (IQR); categorical variables are presented as n (%). Paired weight and dose analyses used only patients with both baseline and follow-up values. Within-patient comparisons used two-sided paired t-tests when distributions were approximately symmetric and Wilcoxon signed-rank tests otherwise; formal normality testing was not used. No multiplicity adjustment was applied because analyses were exploratory and descriptive. Week-12 HbA1c target attainment was calculated only among the 498 patients with a week-12 HbA1c measurement; paired baseline–week-12 HbA1c change was summarized among the 480 patients with both measurements. HbA1c values ≥ 20% were considered implausible and excluded (3 baseline and 2 week-12 records). A multivariable logistic regression model examined the exploratory outcome of starting dose >15 U. The model included age (per 10 years), sex, study period, and mutually exclusive prior-treatment pathway; reference categories were female sex, T1, and non-injectable therapy. All model covariates were available for the 1499 participants. Adjusted odds ratios (ORs), 95% confidence intervals (CIs), and P values are reported in Supplementary Table 3. The >15-U threshold was a pragmatic exploratory cutoff used to distinguish relatively higher observed starting doses; it was not outcome-derived or a regulatory, product-label, guideline, or treatment-decision threshold. Post hoc exploratory ordinary least-squares models estimated crude and adjusted mean differences in baseline-to-follow-up change across prior-treatment pathways. Non-injectable therapy was the reference. The adjusted HbA1c model included baseline HbA1c, age, sex, and baseline BMI; the adjusted body-weight model included baseline body weight, age, and sex. HC3 robust standard errors were used, and pathway terms were assessed jointly with a Wald test. Results are reported as mean differences in change with 95% CIs and P values. The insulin-plus-GLP-1RA pathway was retained in descriptive summaries but excluded from these inferential models because only 4 paired HbA1c and 7 paired body-weight observations were available. Adjusted models used complete cases, and no multiplicity adjustment was applied. These models estimate associations and were not interpreted as causal comparative effectiveness. Center identifiers were unavailable in the cleaned database, so site-level clustering could not be modeled.

## 3 Results

### 3.1 Patient baseline characteristics and treatment pathway distribution

The study cohort comprised 1499 patients with at least 3 months of documented continuous IDegLira use and available observation (165.33 ± 44.97 days), classified into five mutually exclusive prior-treatment pathways as defined in the Methods section: 591 non-injectable therapy, 384 basal insulin, 301 MDI, 139 GLP-1RA-based therapy, and 84 insulin plus GLP-1RA.

### 3.2 IDegLira dose practice: observed starting-dose variation by prior treatment pathway

The mean starting dose differed across prior-treatment pathways, ranging from 12.73 U in the non-injectable pathway to 16.05 U in the insulin-plus-GLP-1RA pathway (Fig. 1A). These are observed group-level differences and do not demonstrate patient-level individualized dosing or an optimal dosing algorithm (see Table 1) (see Fig. 2).

**Fig. 1:**
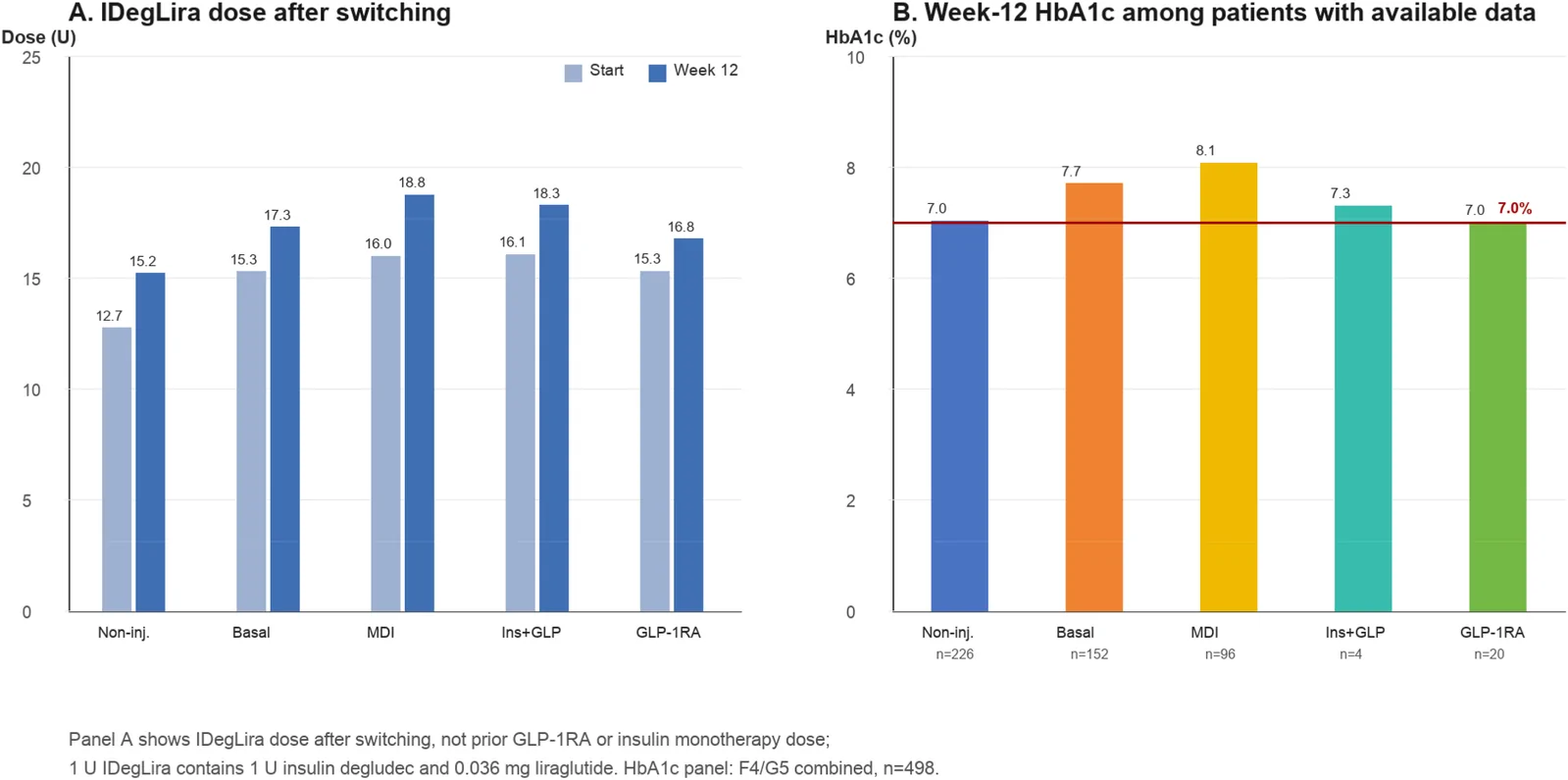
IDegLira dose and week-12 HbA1c outcomes by prior treatment pathway (A) IDegLira starting and week-12 dose after switching, by prior treatment pathway. IDegLira dose is reported in U and is not a prior GLP-1RA or insulin monotherapy dose; 1 U IDegLira contains 1 U insulin degludec and 0.036 mg liraglutide. (B) Week-12 HbA1c among patients with available data. Data are mean values. HbA1c panel uses the F4/G5 combined week-12 HbA1c field (n = 498). T3-only paired body-weight and BMI changes (n = 487 and n = 465, respectively) are summarized in Supplementary Table 4. GLP-1RA, glucagon-like peptide-1 receptor agonist; MDI, multiple daily injections.

**Fig. 2:**
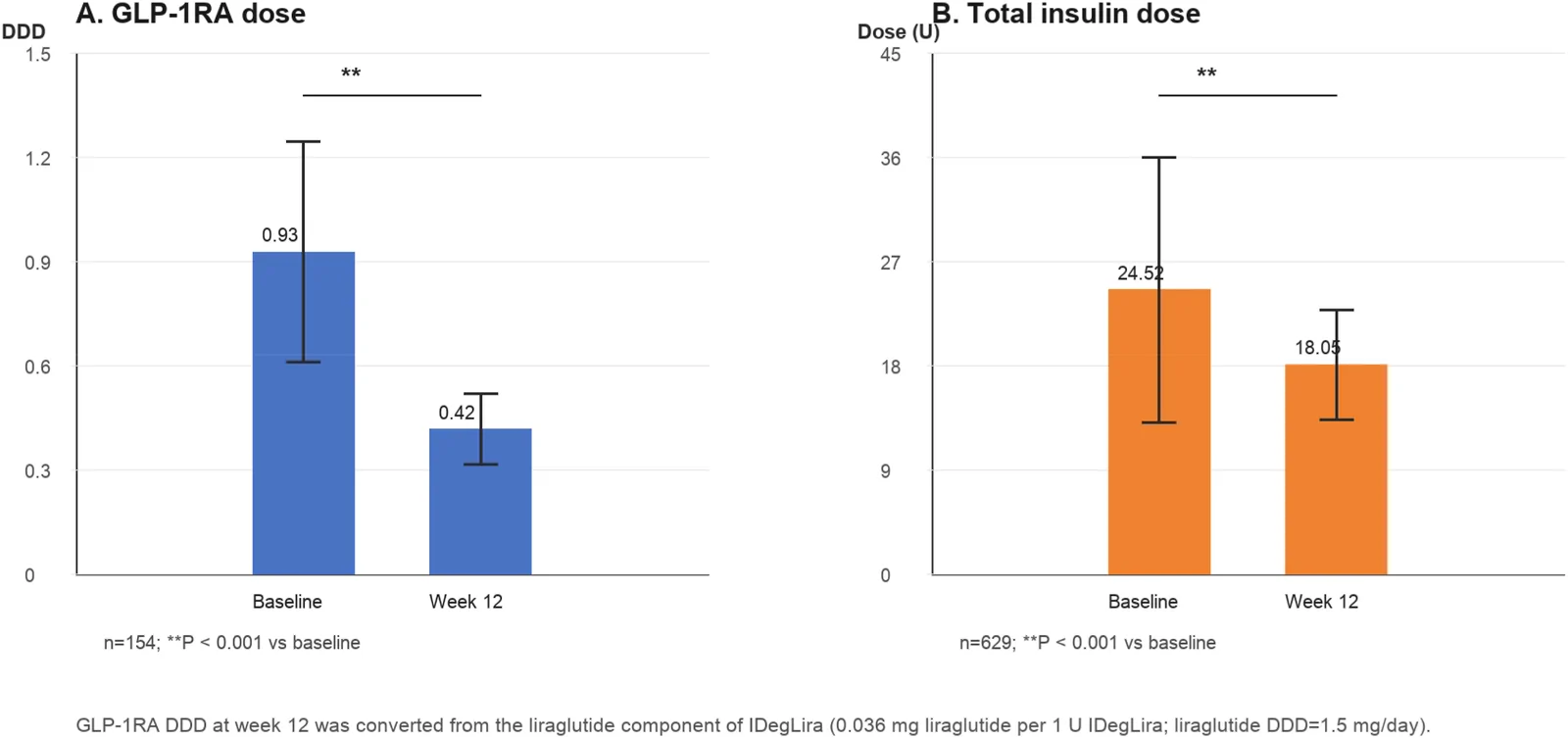
Changes in total insulin and GLP-1RA dose from baseline to week 12. (A) GLP-1RA dose (DDD); (B) total insulin dose (U). Data are mean ± SD among patients with paired baseline and week-12 dosing data. **P < 0.001 vs baseline. DDD, defined daily dose. Baseline DDD represented separately prescribed GLP-1RA; week-12 DDD was converted from the liraglutide component of IDegLira and was not additionally counted as separate concomitant GLP-1RA therapy.

The starting dose had a median of 14 U (IQR 10–16), a mean of 14.46 ± 4.06 U, and a range of 6–40 U. Starting doses ≤20 U accounted for 95.60% of the cohort; starting doses ≥30 U and exactly 40 U accounted for 0.73% and 0.07%, respectively (Table 2). The isolated 40-U value was the maximum observed starting dose, not a protocol maximum or recommended starting dose.

**Table 1:** Patient baseline characteristics and IDegLira dosing parameters.

	Overall	Oral/non-injectable	Basal insulin	MDI	Insulin + GLP-1RA	GLP-1RA
FAS, N	1499 (100.00%)	591 (39.43%)	384 (25.62%)	301 (20.08%)	84 (5.60%)	139 (9.27%)
Male, n (%)	908 (60.57%)	364 (61.59%)	221 (57.55%)	198 (65.78%)	46 (54.76%)	79 (56.83%)
Age, years	54.76 ± 10.06	51.59 ± 10.02	57.15 ± 8.00	58.23 ± 10.72	54.40 ± 9.46	54.38 ± 10.04
Weight, kg	75.55 ± 11.19	77.80 ± 11.25	72.68 ± 11.26	74.31 ± 10.64	75.01 ± 11.47	77.24 ± 9.53
BMI, kg/m^2^	26.76 ± 3.25	27.63 ± 3.32	25.93 ± 3.01	26.18 ± 3.24	26.29 ± 3.46	27.13 ± 2.48
Baseline HbA1c, % (available cases; n = 1374 overall)	9.05 ± 1.78	9.17 ± 1.70	9.03 ± 1.98	9.11 ± 2.02	8.30 ± 0.84	8.81 ± 1.04
FPG, mmol/L (available cases)	9.75 ± 2.01	10.01 ± 2.20	9.74 ± 1.74	9.50 ± 1.99	8.84 ± 1.47	9.79 ± 1.97
PPG, mmol/L (available cases)	14.06 ± 4.76	14.33 ± 2.80	14.16 ± 2.89	13.58 ± 3.68	12.99 ± 2.75	14.49 ± 12.38
Starting IDegLira dose, U	14.46 ± 4.06	12.73 ± 3.16	15.28 ± 3.62	15.98 ± 5.28	16.05 ± 3.37	15.29 ± 3.15
Week-12/current IDegLira dose, U (available cases; n = 1306 overall)	16.72 ± 4.55	15.19 ± 3.91	17.28 ± 4.19	18.75 ± 5.35	18.27 ± 4.27	16.75 ± 3.98

Baseline characteristics and IDegLira dosing parameters of 1499 patients with T2D stratified by five mutually exclusive prior-treatment pathways. Data are presented as mean ± SD or n (%), based on available cases. HbA1c, FPG, PPG, and follow-up-dose rows have endpoint-specific denominators; paired baseline–week-12 HbA1c changes (n = 480) are reported in Supplementary Table 8, and exploratory crude and adjusted between-pathway associations are reported in Supplementary Table 9. BMI, body mass index; FPG, fasting plasma glucose; GLP-1RA, glucagon-like peptide-1 receptor agonist; MDI, multiple daily injections; PPG, postprandial plasma glucose.

**Table 2:** IDegLira starting dose distribution.

Starting dose, U	Number of patients at baseline	Proportion of patients at baseline (%)
FAS, N	1499	100.00
≤ 10 U	390	26.02
>10 to≤15 U	389	25.95
>15 to≤20 U	654	43.63
>20 to≤25 U	35	2.33
>25 to≤30 U	27	1.80
>30 to≤40 U	4	0.27

IDegLira starting dose distribution. Data are presented as n (%).

Among 1306 patients with available week-12/current doses, the median was 16 U (IQR 14–20) and the mean was 16.72 ± 4.55 U, compared with a mean starting dose of 14.46 ± 4.06 U in all 1499 patients. Among patients with both measurements, the mean dose change was +2.40 ± 3.63 U (n = 1306). Each 1 U of IDegLira contains 1 U insulin degludec and 0.036 mg liraglutide. The adjusted regression results are reported in Supplementary Table 3.

### 3.3 Glycemic control and weight changes by prior treatment pathway

Observed week-12 HbA1c status, body-weight change, regimen simplification, and dose changes were summarized by prior-treatment pathway. The endpoint denominators differed: week-12 HbA1c n = 498, paired baseline–week-12 HbA1c n = 480, paired body weight n = 487 (T3 only), paired insulin dose n = 629, and paired GLP-1RA dose n = 154. Among the 480 patients with paired baseline and week-12 HbA1c data (all enrolled in the final study period), mean HbA1c decreased from 9.11 ± 2.09% to 7.45 ± 2.28% (mean change −1.66 ± 2.23 percentage points; 95% CI −1.86 to −1.46; P < 0.001 by paired *t*-test and Wilcoxon signed-rank test). Decreases were observed across prior-treatment pathways (non-injectable therapy −2.09, basal insulin −1.34, MDI −1.09, and GLP-1RA −2.31 percentage points; all P < 0.001; Supplementary Table 8). In the adjusted complete-case model (n = 422; insulin plus GLP-1RA excluded because n = 4), the pathway terms were jointly associated with HbA1c change (global P < 0.001). Relative to non-injectable therapy, adjusted mean differences were +0.82 percentage points (95% CI 0.37 to 1.27) for basal insulin, +1.25 (95% CI 0.53 to 1.98) for MDI, and −0.08 (95% CI −0.45 to 0.29) for GLP-1RA-based therapy; positive values indicate smaller reductions (Supplementary Table 9). In the evaluable-versus-non-evaluable comparison (Supplementary Table 6), patients with week-12 HbA1c data were older (56.23 ± 9.83 vs 54.03 ± 10.11 years; P < 0.001), had higher baseline BMI (27.14 ± 3.22 vs 26.55 ± 3.25 kg/m^2^; P = 0.002), and differed in prior-treatment pathway distribution (P < 0.001); all evaluable patients were enrolled in T3. Sex, baseline FPG, and baseline PPG did not differ significantly; within T3, baseline HbA1c also did not differ between evaluable and non-evaluable patients (9.11 ± 2.09% vs 9.20 ± 1.84%; P = 0.641).

Among patients with available week-12 HbA1c data (n = 498), mean HbA1c was 7.44 ± 2.29% and the median was 7.0% (IQR 6.7–7.2). The proportions with HbA1c ≤ 6.5%, <7.0%, <7.5%, and <8.0% were 21.08%, 47.39%, 81.73%, and 89.36%, respectively. Baseline HbA1c was 9.05 ± 1.78% among patients with available baseline HbA1c data (n = 1374). Because paired baseline and week-12 HbA1c data were not available for all patients, threshold attainment is presented descriptively for the week-12 evaluable population.

Six-point self-monitored plasma glucose (SMPG) profiles decreased significantly from baseline to week 12 (Supplementary Fig. 1). Fasting and postprandial glucose decreased significantly over the same period (fasting: 2.95 ± 1.96 mmol/L; breakfast: 4.75 ± 7.02 mmol/L; lunch: 4.42 ± 3.02 mmol/L; dinner: 4.31 ± 3.58 mmol/L; overall: 4.39 ± 3.08 mmol/L; all P < 0.001).

In the final study period (T3), paired body-weight data were available for 487 patients. Mean body weight decreased from 75.48 ± 11.12 kg at baseline to 72.72 ± 10.56 kg at follow-up (mean change −2.76 ± 4.93 kg; P < 0.001 by paired *t*-test and Wilcoxon signed-rank test). Paired BMI data were available for 465 patients, with a mean change of −1.03 ± 1.43 kg/m^2^. By prior-treatment pathway, mean body-weight changes were −3.15 ± 4.86 kg (n = 192) for non-injectable therapy, −2.17 ± 3.11 kg (n = 157) for basal insulin, −3.25 ± 4.16 kg (n = 107) for MDI, +6.43 ± 22.30 kg (n = 7) for insulin plus GLP-1RA, and −4.04 ± 3.11 kg (n = 24) for GLP-1RA-based therapy. These are unadjusted T3-only descriptive findings. In the crude inferential model excluding the sparse insulin-plus-GLP-1RA pathway (n = 480), the global pathway P value was 0.007. After adjustment for baseline body weight, age, and sex, there was no overall pathway association with body-weight change (global P = 0.606); adjusted mean differences versus non-injectable therapy were +0.07 kg (95% CI −0.59 to 0.72) for basal insulin, −0.54 kg (95% CI −1.49 to 0.40) for MDI, and −0.22 kg (95% CI −1.77 to 1.34) for GLP-1RA-based therapy (Supplementary Table 9).

### 3.4 Observed outcome patterns across prior-treatment pathways

Based on clinical data available after the minimum 3-month continuous-use period from patients in each treatment pathway, we summarized observed outcome patterns across clinically relevant domains, including week-12 glycemic status among evaluable patients, body-weight change, regimen simplification, and insulin dose adjustment. Because complete paired HbA1c and scoring-source data were not consistently available across all pathways, no composite efficacy score was used.

Observed clinical outcomes differed across prior-treatment pathways. Among patients with available week-12 HbA1c, the non-injectable pathway had a mean HbA1c of 7.03 ± 0.97% (n = 226) and the basal-insulin pathway 7.70 ± 2.87% (n = 152); these are cross-sectional evaluable-population summaries. Paired body-weight changes were −3.15 ± 4.86 kg (n = 192) and −2.17 ± 3.11 kg (n = 157), respectively, in the T3-only paired-weight population. Exploratory crude and adjusted between-pathway estimates for paired HbA1c and body-weight change are reported in Supplementary Table 9. In the MDI pathway, prandial-insulin discontinuation was recorded in 269/301 patients (89.37%); concurrent paired HbA1c and systematic post-initiation safety data were not available for this subgroup.

The descriptive summaries and exploratory models show different observed outcome patterns across prior-treatment pathways. These associations do not establish that any prior-treatment pathway or the subsequent switch caused superior outcomes.

### 3.5 Exploratory conceptual framework for IDegLira treatment-transition patterns

Fig. 3 presents an exploratory conceptual framework that synthesizes observed pathway-specific patterns. It is hypothesis-generating, has not been prospectively developed or externally validated, and should not be interpreted as a clinical decision algorithm or a recommendation for a specific starting dose.

**Fig. 3:**
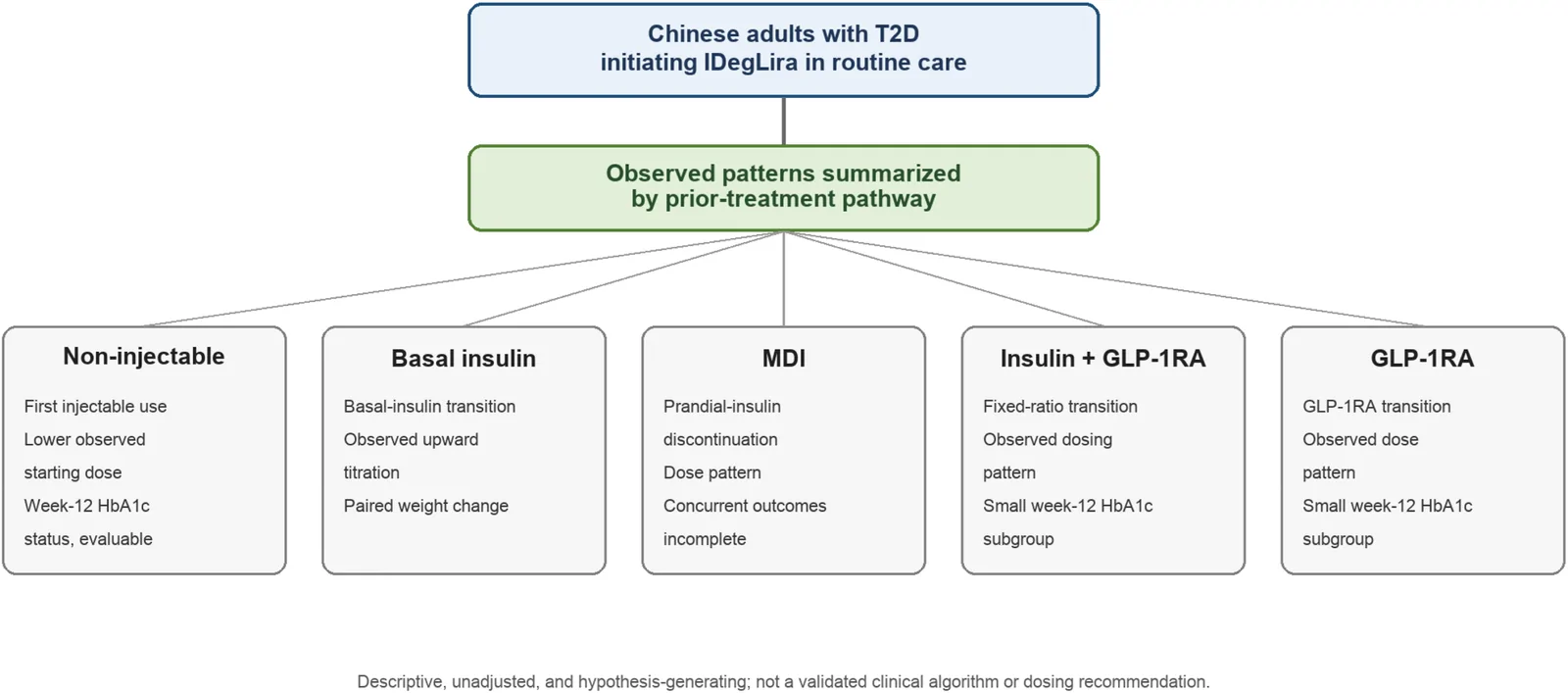
Exploratory conceptual framework for observed IDegLira treatment-transition patterns Exploratory conceptual framework synthesizing observed pathway-specific patterns by prior therapy, including week-12 HbA1c status among evaluable patients, body-weight/BMI change, dosing patterns, and regimen simplification. The framework is hypothesis-generating, has not been prospectively developed or externally validated, and should not be interpreted as prescriptive clinical guidance.

### 3.6 Concomitant medication changes

Among 1022 patients using at least one oral glucose-lowering drug at baseline, 540 discontinued one or more oral agents; this corresponds to 36.02% of the full cohort (540/1499) and 52.84% of the 1022 baseline oral-drug users. Specific oral-drug classes were not consistently available for all source periods. Among patients with paired GLP-1RA-dose data (n = 154), baseline DDD represented separately prescribed GLP-1RA, whereas follow-up DDD was converted from the liraglutide component contained in IDegLira. The observed difference therefore reflects a change in recorded GLP-1RA dose/formulation and cannot establish complete GLP-1RA discontinuation or reduced total GLP-1RA exposure; liraglutide delivered within IDegLira was not additionally counted as separate concomitant GLP-1RA therapy. Among patients with paired insulin-dose data (n = 629), total insulin dose changed from 24.52 ± 11.44 to 18.05 ± 4.76 U (P < 0.001).

### 3.7 Temporal patterns in insurance coverage and prescribing

Insurance coverage and prescribing patterns changed over the three observation periods. T1 (April–August 2022), T2 (May–June 2023), and T3 (November–December 2023) were predefined source-data periods with different recruitment windows and cohort sizes. T1 and T3 comparisons are descriptive and were not adjusted for period size, center, patient mix, or other time-varying factors. The observed changes coincided temporally with reimbursement inclusion but cannot be attributed causally to reimbursement.

## 4 Discussion

This multicenter real-world registry describes IDegLira treatment-transition pathways in Chinese adults with T2D. Its principal contribution is descriptive: observed starting-dose and available follow-up-dose patterns, outcome patterns, and regimen-simplification patterns are reported across five mutually exclusive prior-treatment pathways. The proposed pathway framework is exploratory and hypothesis-generating, not validated clinical guidance.

Patients transitioning from different prior-treatment pathways demonstrated different observed patterns of glycemic status, weight change, and regimen simplification. In exploratory adjusted analyses, HbA1c change was associated with prior pathway, whereas body-weight change was not. These estimates are vulnerable to residual and unmeasured confounding and should not be interpreted as pathway superiority or as effects of switching to IDegLira.

Among MDI users, prandial-insulin discontinuation was recorded in 269/301 patients (89.37%) during the available follow-up period. Regimen simplification after switching from basal-bolus insulin has also been described in the prospective REX study and the retrospective STOP study [29,30]. However, those studies included longer follow-up and longitudinal glycemic and/or hypoglycemia assessments. Our finding therefore describes documented treatment simplification only; it does not establish maintained glycemic control, reduced hypoglycemia, improved adherence, or long-term persistence because concurrent paired HbA1c, systematic safety, and patient-reported data were unavailable for this subgroup.

Among patients previously receiving basal insulin, the paired HbA1c analysis showed a mean within-patient change of −1.34 percentage points (n = 146; Supplementary Table 8), and the T3 paired-weight analysis showed a mean change of −2.17 ± 3.11 kg (n = 157; Supplementary Table 4). In the exploratory HbA1c model, the adjusted mean difference versus non-injectable therapy was +0.82 percentage points (95% CI 0.37 to 1.27), indicating a smaller observed reduction; the adjusted body-weight difference was +0.07 kg (95% CI −0.59 to 0.72). Because pathway assignment was not randomized and no untreated or alternative-treatment comparator was available, these associations do not establish that the prior pathway or switching caused the outcomes.

Starting doses varied across prior-treatment pathways, and the regression model found associations with age and prior treatment pathway. The underlying reasons for individual prescribing decisions, including physician rationale, treatment goals, hypoglycemia concerns, and resource constraints, were not directly collected. These explanations are therefore hypotheses rather than findings established by this study.

The observed week-12/current dose and HbA1c distribution differed from DUAL II China [32], but comparisons between this registry and a randomized trial are confounded by differences in eligibility, baseline HbA1c, prior treatment, titration algorithms, follow-up intensity, adherence, and endpoint definitions. A recent Chinese retrospective database cohort also reported 180-day glycemic, insulin-dose, persistence, and safety outcomes across prior-treatment subgroups [28], but its database design, 180-day effectiveness population, and endpoint ascertainment differ from those of the present registry. In our evaluable week-12 population, 47.4% had HbA1c <7%. In the paired population (n = 480), mean HbA1c decreased by 1.66 percentage points (P < 0.001); although consistent with glucose lowering, this within-patient change cannot be causally attributed to IDegLira in the absence of a comparator group.

Temporal changes in insurance coverage, patient mix, and dose patterns coincided with the reimbursement period. Because no causal or adjusted policy analysis was performed, these observations cannot establish that reimbursement improved access or changed physician dosing decisions.

Changes in recorded oral or separately prescribed GLP-1RA therapy may reflect regimen simplification, duplication avoidance, physician preference, reimbursement, tolerability, or glycemic response. They should not be interpreted as direct pharmacological benefits. For the GLP-1RA analysis, baseline DDD represented separately prescribed GLP-1RA and follow-up DDD was converted from the liraglutide component of IDegLira; therefore, the difference does not establish discontinuation or reduced total GLP-1RA exposure. Liraglutide contained in IDegLira was not counted as separate concomitant GLP-1RA treatment.

Several limitations are important. First, baseline HbA1c was available for 1374/1499 patients, week-12 HbA1c for 498/1,499, and paired baseline–week-12 HbA1c for 480 patients. Standardized week-12 HbA1c collection was introduced in T3 and was not included in the T1/T2 questionnaires; all paired observations therefore came from T3. Individual-level reasons for absent measurements within T3 were not systematically recorded. Separately, three baseline and two week-12 HbA1c values ≥ 20% were considered implausible and excluded as a data-quality rule. Second, the study had no untreated or alternative-treatment comparator group, so changes cannot be attributed specifically to IDegLira rather than treatment intensification, concomitant-medication changes, regression to the mean, lifestyle, or increased follow-up. Third, the exploratory pathway models adjusted only for recorded baseline covariates and remain subject to residual and unmeasured confounding. The predefined site and physician eligibility criteria may also limit representativeness of healthcare settings and clinicians. Fourth, the minimum documented continuous IDegLira exposure and follow-up was 3 months (approximately 12 weeks), and no comprehensive post-initiation hypoglycemia, adverse-event, serious-adverse-event, or patient-reported outcome dataset was available across all periods. This exposure requirement may underrepresent early discontinuers and limit generalizability. Fifth, the proposed framework was not prospectively developed or externally validated. Finally, temporal reimbursement associations were descriptive. Longer comparative studies with standardized HbA1c collection, safety monitoring, patient-reported outcomes, and center-aware analyses are needed.

## 5 Conclusions

In summary, this multicenter retrospective observational registry describes real-world IDegLira treatment-transition and dosing patterns across five prior-treatment pathways. Glycemic findings were limited to the evaluable follow-up population (498 of 1499 patients, all from T3); paired baseline–week-12 HbA1c data (n = 480) showed a mean reduction of 1.66 percentage points. In the MDI pathway, prandial-insulin discontinuation was documented in 269/301 patients (89.37%), describing regimen simplification without establishing maintained glycemic control or a safety benefit. Exploratory pathway associations were hypothesis-generating; the study had no untreated or alternative-treatment comparator group and no basis for causal inference. These findings provide descriptive real-world evidence that may inform clinical discussions and generate hypotheses for future comparative studies.

## CRediT authorship contribution statement

**Jie Zhang:** Writing – review & editing, Writing – original draft, Data curation. **Weihao Wang:** Formal analysis, Data curation. **Fuli Man:** Investigation, Data curation. **Xiaoxia Wang:** Data curation. **Qi Pan:** Resources, Data curation, Conceptualization. **Lixin Guo:** Conceptualization.

## Ethics approval and consent to participate

The study protocol (No. 2023BJYYEC-087-01) was approved by the Ethics Committee of Beijing Hospital on May 3, 2023. The requirement for written informed consent was waived by the Ethics Committee because this was a non-interventional observational study using routine clinical data.

## Study registration

This non-interventional observational study was retrospectively registered with the Chinese Clinical Trial Registry (ChiCTR2500096325) for transparency. All treatment decisions, including IDegLira initiation and titration, were made by treating physicians as part of routine clinical practice, and no intervention was assigned by the investigators.

## Declaration of generative AI and AI-assisted technologies in the writing process

AI-assisted tools were used for limited editorial support. The authors reviewed the final manuscript and take full responsibility for its content.

## Funding

No financial funding was received for this study.

## Declaration of competing interest

The authors declare that they have no known competing financial interests or personal relationships that could have appeared to influence the work reported in this paper.
