# Supplementary material for: Real-world treatment-transition pathways and dosing patterns of insulin degludec/liraglutide in Chinese adults with type 2 diabetes
Source: Metabol Open. 2026 Sep 15;32:100497. doi: 10.1016/j.metop.2026.100497 (PMC13651640; doi:10.1016/j.metop.2026.100497)
Supplement: Supplementary file 1 [file mmc1.docx]

**Supplementary Table 1. Clinical Parameters of Patients Entering the Study Across Three Observation Periods**

|  | **T1 (2022.4-8)** | **T2 (2023.5-6)** | **T3 (2023.11-12)** |
| --- | --- | --- | --- |
| IDegLira entered the national medical insurance | No | Yes | Yes |
| FAS, N | 257 (17.14%) | 571 (38.09%) | 671 (44.76%) |
| Health care fee, n (%) |  |  |  |
| Medical insurance | 194 (12.94%) | 558 (37.22%) | 660 (44.03%) |
| Self-paid | 63 (4.20%) | 13 (0.87%) | 11 (0.73%) |
| Previous treatment regimen, n (%) |  |  |  |
| Non-injectable therapy | 50 (3.34%) | 241 (16.08%) | 300 (20.01%) |
| Basal insulin | 51 (3.40%) | 134 (8.94%) | 199 (13.28%) |
| MDI | 51 (3.40%) | 113 (7.54%) | 137 (9.14%) |
| Insulin+GLP-1RA | 53 (3.54%) | 24 (1.60%) | 7 (0.47%) |
| GLP-1RA | 52 (3.47%) | 59 (3.94%) | 28 (1.87%) |
| Dose of IDegLira, U |  |  |  |
| Starting dose | 15.18 ± 3.62 | 14.46 ± 4.41 | 14.18 ± 3.86 |
| Dose at 12 weeks | 15.98 ± 4.26 | 16.42 ± 4.34 | 17.09 ± 4.73 |

**Supplementary Table 2. Research-Site and Physician Selection Profile Across Three Recruitment Periods**

| **Recruitment period** | **Secondary hospitals** | **Tertiary hospitals** | **Total hospitals** | **Secondary share** | **Tertiary share** |
| --- | --- | --- | --- | --- | --- |
| T1 | 42 | 158 | 200 | 21.0% | 79.0% |
| T2 | 66 | 199 | 265 | 24.9% | 75.1% |
| T3 | 68 | 203 | 271 | 25.1% | 74.9% |
| Three-period total | 176 | 560 | 736 | 23.9% | 76.1% |

Note: All selected hospitals were secondary-level or tertiary-level institutions. The site-selection plan maintained an urban-to-county ratio of 2:1. Participating physicians were senior attending physicians or above, and no physician contributed more than three enrolled patients. Patients included in the registry had documented continuous use of once-daily IDegLira (the fixed-ratio combination [FRC]) for at least 3 months.

**

**

**

**

**

**

Change of blood glucose(mmol/L)

|  | Baseline | 12 weeks |
| --- | --- | --- |
| FBG | 9.67002 | 6.78776 |
| PBG1 | 14.0734 | 9.48935 |
| PBG2 | 13.653 | 9.57427 |
| PBG3 | 13.3798 | 9.50186 |
| BBG | 11.1266 | 8.2116 |
| NBG | 9.34085 | 7.10517 |

**Supplementary Figure 1. Changes in six-point self-monitored plasma glucose from baseline to week 12**

Fasting and postprandial glucose levels at baseline and week 12. Data are mean ± SD. P < 0.001 vs baseline. FBG, fasting blood glucose; PBG, postprandial blood glucose; PBG1, breakfast; PBG2, lunch; PBG3, evening meal; BBG, bedtime blood glucose; NBG, nocturnal blood glucose.

**Supplementary Table 3. Multivariable Logistic Regression for Starting Dose >15 U**

| **Factor** | **OR** | **95% CI** | **P value** |
| --- | --- | --- | --- |
| Age, per 10 years | 1.20 | 1.07-1.35 | 0.002 |
| Male vs female | 1.06 | 0.85-1.33 | 0.604 |
| T2 vs T1 | 0.94 | 0.67-1.30 | 0.698 |
| T3 vs T1 | 0.78 | 0.56-1.09 | 0.139 |
| Basal insulin | 4.04 | 3.05-5.36 | <0.001 |
| MDI | 3.59 | 2.65-4.87 | <0.001 |
| Insulin+GLP-1RA | 5.91 | 3.45-10.13 | <0.001 |
| GLP-1RA | 3.86 | 2.58-5.76 | <0.001 |

The model included age (per 10 years), sex, observation period, and mutually exclusive prior-treatment pathway. Reference category: female participants in T1 with non-injectable therapy. The model included all 1,499 participants. OR, odds ratio; CI, confidence interval.

**Supplementary Table 4. Body-Weight and BMI Changes from Baseline to Week 12 by Prior-Treatment Pathway**

| **Prior treatment pathway** | **Paired n (weight / BMI)** | **Body weight, kg: baseline → week 12; change** | **BMI, kg/m²: baseline → week 12; change** |
| --- | --- | --- | --- |
| Overall (T3 only) | 487 / 465 | 75.48 ± 11.12 → 72.72 ± 10.56; −2.76 ± 4.93 | 26.84 ± 3.26 → 25.80 ± 2.93; −1.03 ± 1.43 |
| Non-injectable therapy | 192 / 180 | 76.96 ± 10.93 → 73.82 ± 9.76; −3.15 ± 4.86 | 27.51 ± 3.37 → 26.33 ± 2.96; −1.17 ± 1.72 |
| Basal insulin | 157 / 154 | 72.16 ± 11.37 → 69.99 ± 10.49; −2.17 ± 3.11 | 25.89 ± 2.98 → 25.07 ± 2.68; −0.82 ± 1.08 |
| MDI | 107 / 102 | 76.03 ± 10.86 → 72.78 ± 10.00; −3.25 ± 4.16 | 26.54 ± 3.32 → 25.47 ± 3.10; −1.07 ± 1.41 |
| Insulin+GLP-1RA | 7 / 6 | 80.86 ± 3.39 → 87.29 ± 24.29; +6.43 ± 22.30 | 28.58 ± 1.75 → 27.87 ± 1.70; −0.71 ± 0.06 |
| GLP-1RA | 24 / 23 | 81.42 ± 7.41 → 77.38 ± 7.51; −4.04 ± 3.11 | 28.81 ± 2.01 → 27.49 ± 2.24; −1.31 ± 0.94 |

Data are mean ± SD at baseline and week 12, with mean change, among T3 patients with paired available data. Negative values indicate reductions. The overall paired sample was n = 487 for body weight and n = 465 for BMI. These are unadjusted descriptive changes and should not be interpreted as pathway-specific treatment effects. BMI, body mass index; GLP-1RA, glucagon-like peptide-1 receptor agonist; MDI, multiple daily injections.

**Supplementary Table 5. Endpoint Availability and Analysis Populations**

| **Endpoint** | **N** | **Percent of total cohort** |
| --- | --- | --- |
| Total cohort | 1,499 | 100.00% |
| Baseline HbA1c available | 1,374 | 91.66% |
| Week-12 HbA1c available | 498 | 33.22% |
| Paired baseline/week-12 HbA1c | 480 | 32.02% |
| Paired baseline/week-12 body weight (T3 only) | 487 | 32.49% |
| Paired baseline/week-12 BMI (T3 only) | 465 | 31.02% |
| Baseline FPG available | 1,415 | 94.40% |
| Week-12 FPG available | 719 | 47.97% |
| Baseline PPG available | 1,215 | 81.05% |
| Week-12 PPG available | 519 | 34.62% |
| Starting dose available | 1,499 | 100.00% |
| Week-12/current dose available | 1,306 | 87.12% |

Endpoint-specific available-case denominators in the cleaned database. No imputation was performed. Paired body-weight and BMI analyses used the T3 baseline body-weight field (D4) and current body-weight field (G5), with BMI calculated from the corresponding recorded BMI fields.

**Supplementary Table 6. Comparison of Patients With and Without Week-12 HbA1c Data**

| **Variable** | **Week-12 HbA1c available (n = 498)** | **Week-12 HbA1c unavailable (n = 1,001)** | **P value** |
| --- | --- | --- | --- |
| Men, n (%) | 305 (61.24%) | 603 (60.24%) | 0.750 |
| Age, years | 56.23 ± 9.83 | 54.03 ± 10.11 | 0.0001 |
| Baseline weight, kg | 76.17 ± 11.23 (n = 446) | 75.20 ± 11.16 (n = 809) | 0.144 |
| Baseline BMI, kg/m2 | 27.14 ± 3.22 (n = 435) | 26.55 ± 3.25 (n = 791) | 0.002 |
| Baseline HbA1c, % | 9.11 ± 2.09 (n = 480) | 9.01 ± 1.58 (n = 894) | 0.350 a |
| Baseline FPG, mmol/L | 9.71 ± 2.21 (n = 486) | 9.77 ± 1.90 (n = 929) | 0.592 |
| Baseline PPG, mmol/L | 13.93 ± 2.67 (n = 395) | 14.12 ± 5.49 (n = 820) | 0.418 |
| Starting dose, U | 14.07 ± 3.41 (n = 498) | 14.65 ± 4.33 (n = 1,001) | 0.005 b |
| Week-12/current dose, U | 17.21 ± 4.76 (n = 482) | 16.43 ± 4.40 (n = 824) | 0.003 |
| Prior-treatment pathway, n (%) | Non-injectable therapy 226 (45.38%); basal insulin 152 (30.52%); MDI 96 (19.28%); GLP-1RA 20 (4.02%); insulin + GLP-1RA 4 (0.80%) | Non-injectable therapy 365 (36.46%); basal insulin 232 (23.18%); MDI 205 (20.48%); GLP-1RA 119 (11.89%); insulin + GLP-1RA 80 (7.99%) | <0.001 |
| Observation period, n (%) | T3 (2023.11–12): 498 (100.00%) c | T1 (2022.4–8): 257 (25.67%); T2 (2023.5–6): 571 (57.04%); T3 (2023.11–12): 173 (17.28%) | <0.001 |
| Insurance status, n (%) | After NRDL: 498 (100.00%) c | Before NRDL: 257 (25.67%); After NRDL: 744 (74.33%) | <0.001 |

Baseline characteristics and dosing variables in the week-12 evaluable (n = 498) and non-evaluable (n = 1,001) groups. Values are mean ± SD or n (%). Continuous variables were compared using two-sided Welch's t-tests; categorical variables were compared using chi-square tests. a Baseline HbA1c was recovered for the final study period (T3) from the raw questionnaire field; paired baseline–week-12 HbA1c data were available for 480 patients. HbA1c values ≥20% were considered implausible and excluded (3 baseline and 2 week-12 records); the single value of 85 was verified as a data-entry error and corrected to 8.5. Within the T3 period, baseline HbA1c did not differ between evaluable and non-evaluable patients (9.11 ± 2.09% vs 9.20 ± 1.84%; P = 0.641). b The Wilcoxon rank-sum P value for starting dose was 0.089; all other variables showed consistent results with both tests. c All 498 week-12-evaluable patients were enrolled in the T3 period (November–December 2023), indicating that week-12 HbA1c availability was primarily determined by the data-collection form used in that period; insurance status followed the same pattern (all evaluable patients were enrolled after NRDL inclusion).

**Supplementary Table 7. Week-12 HbA1c Status by Prior-Treatment Pathway**

| **Prior-treatment pathway** | **Cohort N** | **Week-12 HbA1c N** | **Week-12 HbA1c, %** | **≤6.5%, n** | **<7.0%, n** | **<7.5%, n** | **<8.0%, n** |
| --- | --- | --- | --- | --- | --- | --- | --- |
| Non-injectable therapy | 591 | 226 | 7.03 ± 0.97 | 56 | 114 | 184 | 200 |
| Basal insulin | 384 | 152 | 7.70 ± 2.87 | 35 | 74 | 125 | 138 |
| MDI | 301 | 96 | 8.07 ± 3.33 | 13 | 40 | 78 | 83 |
| GLP-1RA | 139 | 20 | 7.00 ± 0.37 | 1 | 8 | 17 | 20 |
| Insulin + GLP-1RA | 84 | 4 | 7.30 ± 0.14 | 0 | 0 | 3 | 4 |

Cross-sectional summaries among patients with available week-12 HbA1c data (n = 498; values ≥20% excluded as implausible). Paired changes are reported in Supplementary Table 8, and exploratory crude and adjusted between-pathway associations are reported in Supplementary Table 9.

**Supplementary Table 8. Paired Baseline-to-Week-12 HbA1c Change by Prior-Treatment Pathway**

| **Prior-treatment pathway** | **Paired n** | **Baseline HbA1c, %** | **Week-12 HbA1c, %** | **Mean change (95% CI), pp** | **P value** |
| --- | --- | --- | --- | --- | --- |
| Overall | 480 | 9.11 ± 2.09 | 7.45 ± 2.28 | −1.66 (−1.86 to −1.46) | <0.001 |
| Non-injectable therapy | 214 | 9.15 ± 1.72 | 7.06 ± 0.97 | −2.09 | <0.001 |
| Basal insulin | 146 | 9.03 ± 2.46 | 7.69 ± 2.82 | −1.34 | <0.001 |
| MDI | 96 | 9.16 ± 2.46 | 8.07 ± 3.33 | −1.09 | <0.001 |
| GLP-1RA | 20 | 9.32 ± 0.76 | 7.00 ± 0.37 | −2.31 | <0.001 |
| Insulin + GLP-1RA | 4 | 8.32 ± 0.57 | 7.30 ± 0.14 | −1.02 | not computed (n = 4) |

Within-patient HbA1c change among patients with paired baseline and week-12 HbA1c data (n = 480, all enrolled in the T3 period). Values are mean ± SD. P values are from two-sided paired t-tests; Wilcoxon signed-rank tests gave consistent results (overall P < 0.001). HbA1c values ≥20% were considered implausible and excluded; the observed value of 85 was verified as a data-entry error and corrected to 8.5. Exploratory between-pathway models are reported in Supplementary Table 9.

**Supplementary Table 9. Exploratory Between-Pathway Associations With Changes in HbA1c and Body Weight**

| **Endpoint / model** | **Comparison vs non-injectable therapy** | **Analysis n (reference / comparison)** | **Mean difference in change (95% CI)** | **P value** | **Global pathway P value** |
| --- | --- | --- | --- | --- | --- |
| HbA1c change / Crude | Basal insulin | 476 (214 / 146) | +0.76 (0.32 to 1.20) | <0.001 | <0.001 |
| HbA1c change / Crude | MDI | 476 (214 / 96) | +1.01 (0.35 to 1.66) | 0.003 |  |
| HbA1c change / Crude | GLP-1RA-based therapy | 476 (214 / 20) | -0.22 (-0.65 to 0.21) | 0.317 |  |
| HbA1c change / Adjusted | Basal insulin | 422 (186 / 131) | +0.82 (0.37 to 1.27) | <0.001 | <0.001 |
| HbA1c change / Adjusted | MDI | 422 (186 / 87) | +1.25 (0.53 to 1.98) | <0.001 |  |
| HbA1c change / Adjusted | GLP-1RA-based therapy | 422 (186 / 18) | -0.08 (-0.45 to 0.29) | 0.681 |  |
| Body-weight change / Crude | Basal insulin | 480 (192 / 157) | +0.98 (0.14 to 1.83) | 0.023 | 0.007 |
| Body-weight change / Crude | MDI | 480 (192 / 107) | -0.11 (-1.16 to 0.94) | 0.842 |  |
| Body-weight change / Crude | GLP-1RA-based therapy | 480 (192 / 24) | -0.90 (-2.34 to 0.55) | 0.225 |  |
| Body-weight change / Adjusted | Basal insulin | 480 (192 / 157) | +0.07 (-0.59 to 0.72) | 0.842 | 0.606 |
| Body-weight change / Adjusted | MDI | 480 (192 / 107) | -0.54 (-1.49 to 0.40) | 0.261 |  |
| Body-weight change / Adjusted | GLP-1RA-based therapy | 480 (192 / 24) | -0.22 (-1.77 to 1.34) | 0.786 |  |

Change was calculated as follow-up minus baseline; negative values indicate reductions. Crude models included pathway only. Adjusted HbA1c models included baseline HbA1c, age, sex, and baseline BMI (n = 422); adjusted body-weight models included baseline body weight, age, and sex (n = 480). All models used ordinary least squares with HC3 robust standard errors; global P values are joint Wald tests of pathway terms. The insulin-plus-GLP-1RA pathway was descriptive only because paired n was 4 for HbA1c and 7 for body weight. No multiplicity adjustment was applied. These post hoc exploratory estimates describe associations and should not be interpreted as causal comparative treatment effects. BMI, body mass index; CI, confidence interval; GLP-1RA, glucagon-like peptide-1 receptor agonist; MDI, multiple daily injections.

**Supplementary Figure 2. Participant and endpoint availability flow**

The diagram shows endpoint availability in the cleaned database. Week-12 HbA1c records were available for 498 patients and baseline HbA1c records for 1,374 patients; paired baseline–week-12 HbA1c values were available for 480 patients (all enrolled in the T3 period). The three lower boxes represent separate endpoint-specific paired analysis populations: body weight (n = 487, T3 only), insulin dose (n = 629), and GLP-1RA dose (n = 154). They are not sequential subsets of the paired HbA1c population.
